# Supplementary material for: The causal relationship between the human gut microbiota and pyogenic arthritis: a Mendelian randomization study
Source: Front Cell Infect Microbiol. 2024 Nov 26;14:1452480. doi: 10.3389/fcimb.2024.1452480 (PMC11629706; doi:10.3389/fcimb.2024.1452480)
Supplement: Supplementary file 1 [file DataSheet1.zip › mendelian test/ebi-a-GCST90027536.csv_forest.pdf]

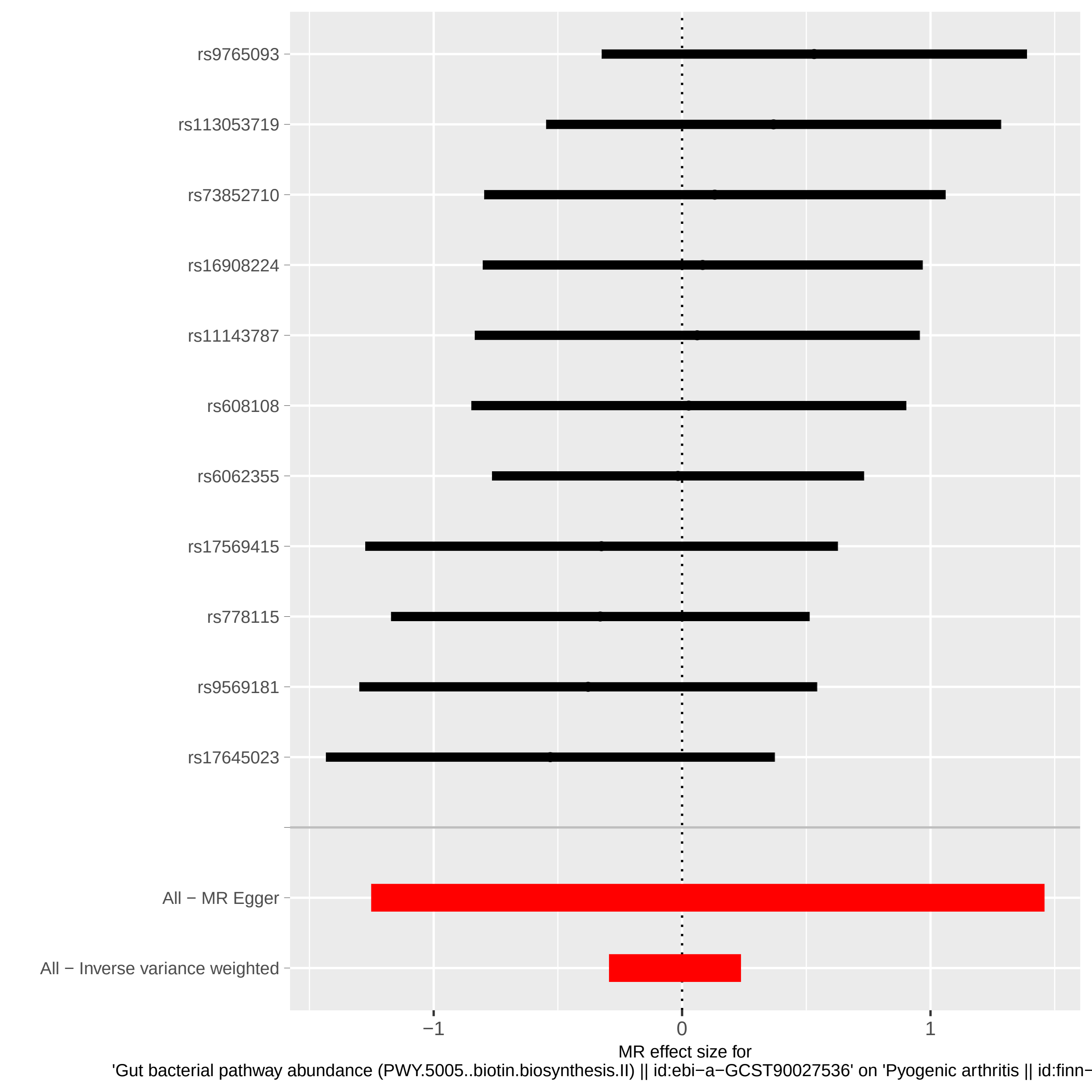
rs9765093
rs113053719
rs73852710
rs16908224
rs11143787
rs608108
rs6062355
rs17569415
rs778115
rs9569181
rs17645023
All − MR Egger
All − Inverse variance weighted
−1
0
1
MR effect size for
'Gut bacterial pathway abundance (PWY.5005..biotin.biosynthesis.II) || id:ebi−a−GCST90027536' on 'Pyogenic arthritis || id:finn−
